# Water-Soluble Copper Ink for the Inkjet Fabrication of Flexible Electronic Components

**DOI:** 10.3390/ma14092218

**Published:** 2021-04-26

**Authors:** Nabi S. Shabanov, Kamil Sh. Rabadanov, Sagim I. Suleymanov, Akhmed M. Amirov, Abdulgalim B. Isaev, Dinara S. Sobola, Eldar K. Murliev, Gulnara A. Asvarova

**Affiliations:** 1 Analytical Center for Collective Use, Dagestan Federal Research Centre of the Russian Academy of Sciences, 367001 Makhachkala, Russia; shabanov.nabi@yandex.ru (N.S.S.); rksh@mail.ru (K.S.R.); s.sagim.i@yandex.ru (S.I.S.); aamirov@mail.ru (A.M.A.); murliev@mail.ru (E.K.M.); konfetka080467@mail.ru (G.A.A.); 2 Department of Inorganic Chemistry and Chemical Ecology, Dagestan State University, St. M. Gadjieva 43-a, Dagestan Republic, 367015 Makhachkala, Russia; abdul-77@yandex.ru; 3 Department of Ceramics and Polymers, Faculty of Mechanical Engineering, Brno University of Technology, Technická 2, 616 69 Brno, Czech Republic; 4 Department of Physics, Faculty of Electrical Engineering and Communication, Brno University of Technology, Technická 2848/8, 616 00 Brno, Czech Republic

**Keywords:** organometallic compound, conductivity, thermogravimetry

## Abstract

The aim of this work is preparation and investigation of copper conductive paths by printing with a different type of functional ink. The solutions based on copper-containing complex compounds were used as inks instead of dispersions of metal nanoparticles. Thermal characteristics of synthesized precursors were studied by thermogravimetry in an argon atmosphere. Based on the comparison of decomposition temperature, the dimethylamine complex of copper formate was found to be more suitable precursor for the formation of copper layers. Structure and performance of this compound was studied in detail by X-ray diffraction, test of wettability, printing on flexible substrate, and electrical measurements.

## 1. Introduction

The use of printing in electronics manufacturing is attracting increasing attention in the field of materials science and technology [1,2,3,4]. Unlike traditional methods of electronics manufacturing, such as vacuum deposition [5], electroplating [6], and photolithography [7], printing technology is an additive approach to electronics manufacturing. This approach consists of the formation of structures by applying functional material to the required area of the substrate, which greatly simplifies the technological process, due to direct printing of the required materials with individual sizes and shapes [8,9]. This makes it possible to manufacture electronic circuits and assemble devices much faster and in a more economical and environmentally friendly way. Moreover, printing technology is compatible with a variety of plastic substrate materials [10], providing electronic devices with additional advantages, such as lightness and flexibility [11].

This technology includes three main stages: preparing materials for printing, printing, and post processing [12]. Conductive materials in particular, which are main parts of electronic devices, are used for printing to create conductive patterns, which provides great prospects for their use [13]. Obviously, only metallic inks, such as gold (Au) [10], silver (Ag) [14], copper (Cu) [15], aluminum (Al) [16], and nickel (Ni) ones [17] are able to provide low resistance within the range from 10 to 100 μOhm·cm. Among them, Ag ink has the highest conductivity. However, it should be noted that along with the market expansion, high cost and scarcity of Ag have hindered its use in large-scale production of printed electronics. As Cu has high conductivity (comparable to that of Ag), but it is much more abundant (1000 times as much as Ag) and much cheaper (about 1% of the cost of Ag), copper ink is gaining increasing attention.

Over the past decade, numerous efforts have been made to develop stable copper paints and highly efficient sintering techniques to create copper patterns at low temperatures and achieve high conductivity and high stability. Although a number of studies have been published on achievements of Cu chemistry and nanotechnology [18,19,20], their authors have mainly focused on synthesis of Cu nanoparticles. A comprehensive review on these materials reliability and stability was recently given by W. Li et.al. [21]. For instance, copper ink composed of 45 nm particles formed layers with resistivity of 17.2 μOhm·cm at 325 °C [22], and the use of particles of about 3.5 nm gave resistivity values of 30.0 μOhm· cm at temperatures above 200 °C [23]. Disadvantages of this type of ink are sedimentation of nanoparticles (ink stability), relatively high probability of clogging of printing head nozzles, and high temperature of subsequent annealing associated with a higher melting point of Cu. However, it is necessary to use a lower temperature, not exceeding 150 °C, to match plastic substrates [24]. In particular, oxidation of the surface of Cu nanoparticles leads to the formation of stable and undesirable oxide layers (Cu_2_O, CuO), which strongly hinder mutual diffusion of Cu atoms between the particles and reduce the formation of percolation paths, and it leads to low conductivity of printed patterns [25]. A solution to the problems described above can be development of inks based on complex compounds, which at relatively low temperatures form functional copper layers directly on the substrate. This paper presents the results obtained in studies of the process of creating copper conductive paths by printing with a different type of functional ink. The purpose of this work is to show the impact of using the solutions based on copper-containing complex compounds, free from disadvantages of dispersions of metal nanoparticles.

## 2. Materials and Methods

### 2.1. Synthesis Procedure

The copper compounds were prepared for their further investigation as precursors in functional inks.

(1) Copper formate Cu (OOCH)_2_ was prepared by adding 5 mL of 85% formic acid to 10 g of copper hydrocarbonate.
(2) Ammonia complex of copper formate [Cu(NH_3_)_2_](OOCH)_2_ was prepared by dissolving 1 g of copper formate Cu(OOCH)_2_ in 2 mL of 25% aqueous ammonia solution.
(3) Dimethylamine complex of copper formate [Cu(C_2_H_6_NH)_2_](OOCH)_2_ was prepared by dissolving 1 g of copper formate Cu(OOCH)_2_ in 2 mL of 33% aqueous solution of dimethylamine.

### 2.2. Characterization Techniques

The analysis of the processes of reduction of copper formate complex-based inks to Cu was performed by X-ray diffraction (XRD) (Shimadzu XRD-6000, Kyoto, Japan) and Raman spectroscopy (Confocal Raman microscope «Senterra» Bruker, Bremen, Germany). Additionally, thermogravimetric analysis was performed on a synchronous thermal analyzer (STA 449 F3 Jupiter “NETZSCH”, Selb, Germany), by examining ink solution samples (100 μL) at a heating rate of 15 °C/min. Elemental analysis was performed by energy-dispersive X-ray spectroscopy (EDX) (ASPEX Express electron microscope equipped by the OmegaMax EDX detector, Sydney, Australia) at accelerating voltage 20 kV.

The films were printed by a commercial inkjet printer EPSON 110 (Epson, Nagano, Japan). Standard piezoelectrically-driven printing head was used with 20 μm diameter of a nozzle. The polyethylene terephthalate (PET) films were chosen as the substrates. The surface of the substrates was processed by an oxygen plasma for increasing of hydrophilicity. The films were printed in the shape of straight strips in numbers from one up to six. These structures were heated in air at the temperatures of 60–160 °C at a temperature-controlled hotplate. The electrical resistance of the prepared films was studied by the standard 2-probe method (Keithley 2002 multimeter). Electrical measurements were carried out for different numbers of the printed passes and for the various processing temperatures. Optical characterization of the printed materials was done by Optical microscope Levenhuk (Levenhuk, Inc., Tampa, FL, USA) after the ink reduction to Cu.

The quantum chemical calculation was carried out by the DFT (density functional theory) method. This method is widely used to calculate the electronic structure of molecules and condensed matter [26,27,28]. The B3LYP approximation is based on a hybrid functional, in which the exchange energy is calculated using the exact result obtained by the Hartree–Fock method. All calculations were performed using the Gaussian 09W program (Gaussian, Inc., Wallingford, CT, USA). The influence of the solvent was not taken into account. The initial structures of the complexes were selected according to the symmetry criteria. At the first stage, the geometry was optimized for each initial configuration at the theoretical level B3LYP/6-31G (d, p), which does not require large computational resources. Further, the stability of each complex was confirmed by the absence of imaginary frequencies in the calculated IR spectra. Further optimization of the geometry and calculation of thermodynamic potentials for the selected complexes was carried out at the level of the B3LYP/6-311++ G (d, p) theory without limitation in symmetry.

## 3. Results and Discussion

### 3.1. Copper-Inks Characterizations

Due to high sensitivity of copper to atmospheric oxygen, the temperature of copper formation from its precursors is of great importance. Therefore, the main requirement for the formation of high-quality copper layers is a low temperature, which is also necessary to ensure the deposition of functional layers on heat-sensitive polymer substrates.

**Figure 1:**
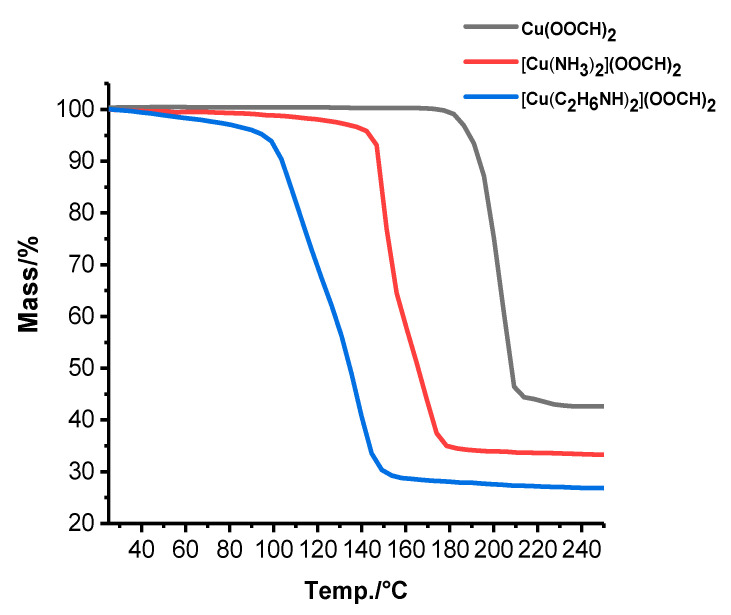
Thermogravimetry of copper precursors.

To determine thermal characteristics, we studied thermogravimetric dependence of synthesized precursors in an argon flow with a heating rate of 10 °C/min. As can be seen from Figure 1, decomposition of copper formate to copper and gaseous compounds takes place at a temperature of 200 °C according to the reaction equation:

Cu(OOCH)_2_ → Cu + CO↑ + CO_2_↑ + H_2_O↑ (1)

The addition of ammonia shifts the decomposition temperature by about 40 °C and a greater shift of 120–140 °C can be observed during decomposition of dimethylamine complex of copper formate, which is already acceptable for the formation of ink on heat-sensitive substrates.

**Figure 2:**
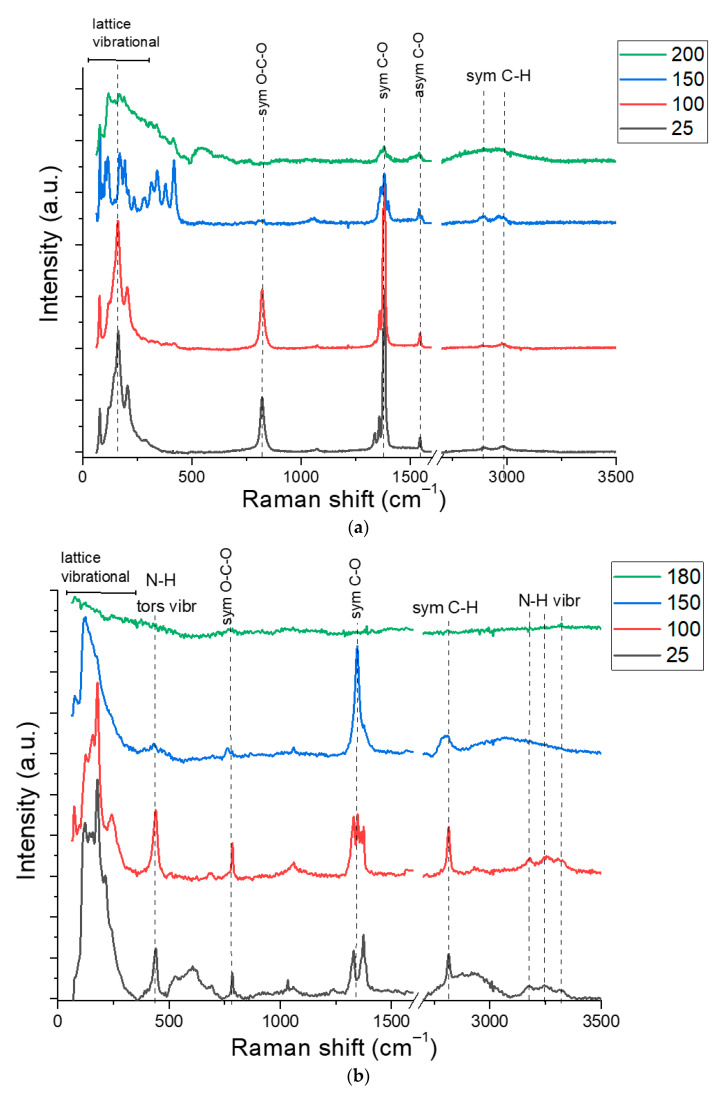

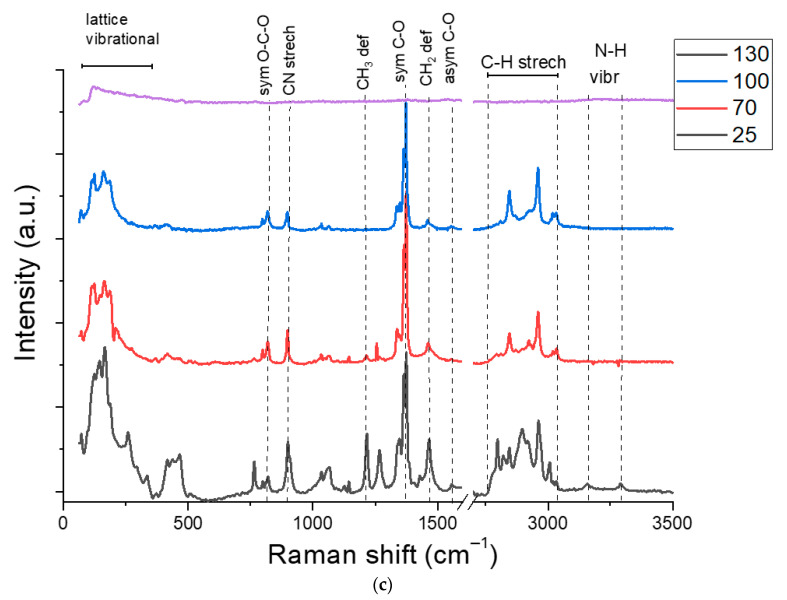
Raman spectra of the samples: (**a**) Cu(OOCH)_2_; (**b**) Cu(NH_3_)_2_](OOCH)_2_; (**c**) [Cu(C_2_H_6_NH)_2_](OOCH)_2_.

Raman spectra were studied to analyze the processes of chemical transformations of copper precursors. Temperature ranges were selected according to the results of thermogravimetric analysis. Raman spectra of copper formate at temperatures of 25–200 °C are presented in Figure 2a. The spectra show characteristic intense bands of stretching vibrations of the formates (1380 cm^−1^ and 826 cm^−1^) [29]. 

Pure copper formate is stable up to 150 °C. The changes at this temperature are observed in the frequency range up to 500 cm^−1^, as well as a decrease of the intensity at 826 cm^−1^ and broadening of the peak at 1380 cm^−1^. We assume, that this is due to a change in the crystal structure and the nature of interactions between the atoms of the copper formate molecules. A decrease in the intensity of all characteristic peaks is observed at 200 °C which indicates a decomposition processes of the sample.

Both characteristic bands and bands of NH group vibrations (3100–3300 cm^−1^) are observed in the Raman spectrum of the ammonia complex of copper formate (Figure 2b). Comparison of the spectra taken at temperatures of 25 and 100 °C show a decrease in the intensity of the background curve. This is due to the evaporation of unbound water and an increase of the crystallinity of the complex compound. The growth of the powder temperature up to 150 °C leads to an increase in the intensity of the symmetric C-O and a decrease in the intensity of symmetric O-C-O and N-H band vibrations. This effect can be associated with the rearrangement of the formate anion and appearance of volatile ammonium formate salts. An increase in temperature up to 180 °C leads to an intensive decomposition of the complex. This is reflected in a decrease in the intensity of all absorption bands of the sample.

Figure 2c shows the Raman spectrum of the complex with dimethylamine. The assignment of vibrations of dimethylamine molecules was carried out according to literature [30]. The spectra of the complex contain bands of symmetric and asymmetric stretching, bending vibrations of CH_2_, CH_3_, and N-H groups. The characteristic bands at 900 cm^−1^ can be attributed to CN vibrations. The absorption bands of the CO carboxyl group at 1380 cm^−1^, O-C-O at 816 cm^−1^ bonds are present. Heating the sample to 70 °C leads to a decrease in the intensity of the broad absorption bands, which are associated with the evaporation of unbound water and growth of the complex crystallinity. A further increase of the temperature up to 100 °C did not change the Raman spectra. However, the release of gaseous reaction products is observed when the temperature was above 110 °C. It leads to the disappearance of the bands of that belong to CN and NH bonds vibrations in the spectra, as well as a decrease in the bands of CH_2_, CH_3_ groups vibrations. Vibrational Raman bands disappear at a temperature of 130 °C. 

**Figure 3:**
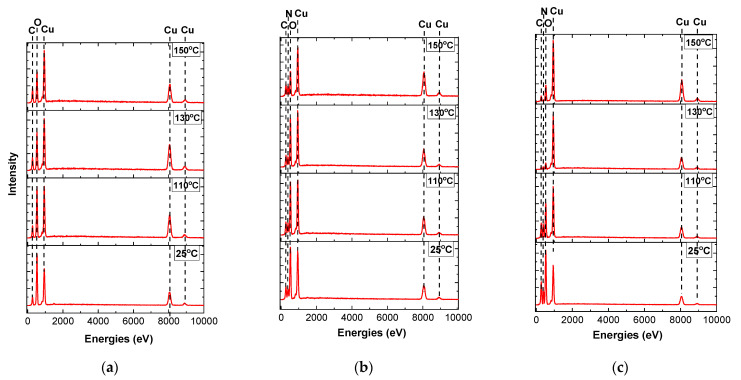
Elemental EDX analysis of the samples after calcination to 150 °C: (**a**) Cu(OOCH)_2_; (**b**) [Cu(NH_3_)_2_](OOCH)_2_; (**c**) [Cu(C_2_H_6_NH)_2_](OOCH)_2_.

Raman spectra show copper reduction temperatures depending on the complexing agent. The data confirm the results of thermogravimetric analysis. However, Raman spectroscopy does not provide information on the content of metallic copper formed as a result of sintering. Therefore, additional studies were carried out by elemental EDX analysis of samples sintered to a 150 °C (Figure 3) accompanied by SEM imaging of the analyzed areas (Table S1). This temperature is considered to be critical for most polymer substrates. Figure 3 shows a relative decrease in the oxygen peak with an increase of the sintering temperature for all three samples. This dependence can be explained by the removal of adsorbed water up to 110–130 °C and by the decomposition of copper formate at higher temperatures. Another indicator of the processes in complex compounds is the change in the nitrogen content in the composition of the samples. Nitrogen is retained after sintering at 150 °C in an amount of 20% in the ammonia complex of copper formate. This is slightly higher than its stoichiometric content in the ammonia complex of the formic acid salt. This indicates the beginning of the decomposition process of the complex with the release of metallic copper. In the complex of copper formate with dimethylamine (Figure 3c), this process is observed at a temperature of 130 °C. In this case, the nitrogen content does not exceed 5%, and the copper content is most evident and reaches 70%. As the temperature rises up to 150 °C, the nitrogen peaks are practically not detected, however, the oxygen content also begins to increase and reaches values of 30%. This can be a result of oxidation of the produced copper.

**Figure 4:**
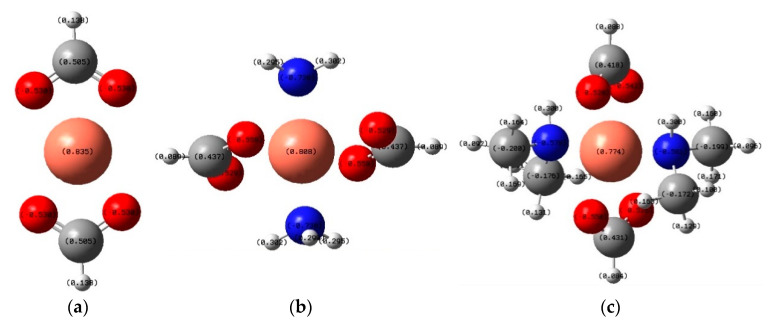
Calculated thermodynamic potentials to estimate the probability of various concurrent reactions for Cu(OOCH)_2_ (**a**), [Cu(NH_3_)_2_](OOCH)_2_ (**b**) and [Cu(C_2_H_6_NH)_2_](OOCH)_2_ (**c**) compounds.

Thus, the results of EDX analysis correlate well with the results of Raman and DTA data and allows us to provide assumptions about the mechanism of copper reduction. A noticeable decrease in the copper reduction temperature depends on the composition of the complexing agent, due to the influence of nitrogen on the copper reduction process, which acts as a bridge for the transfer of an electron from hydrogen of a formate anion to copper, showing, in fact, a catalytic reaction mechanism. To confirm this mechanism, we calculated Mulliken atomic charges and thermodynamic potentials of reactions by the DFT method with the B3LYP/6-311++G(d, p) functional. Figure 4 shows the calculated atomic charges in the Cu(OOCH)_2_ (A), [Cu(NH_3_)_2_](OOCH)_2_ (B) and [Cu(C_2_H_6_NH)_2_](OOCH)_2_ (C) compounds.

As can be seen from the Table 1, the reactions of the formation of complex compounds of a copper cation with ammonia and dimethylamine molecules compete with side reactions of the formation of Cu(OH)_2_, which the oxide CuO produces upon heating, and when a dimethylamine molecule is used as a ligand, the probability of primary reaction (1) increases.

Thus, due to the lowest decomposition temperature, the dimethylamine complex of copper formate is the most preferable precursor for the formation of copper layers; therefore, this compound was chosen as the object of our further research.

**Figure 5:**
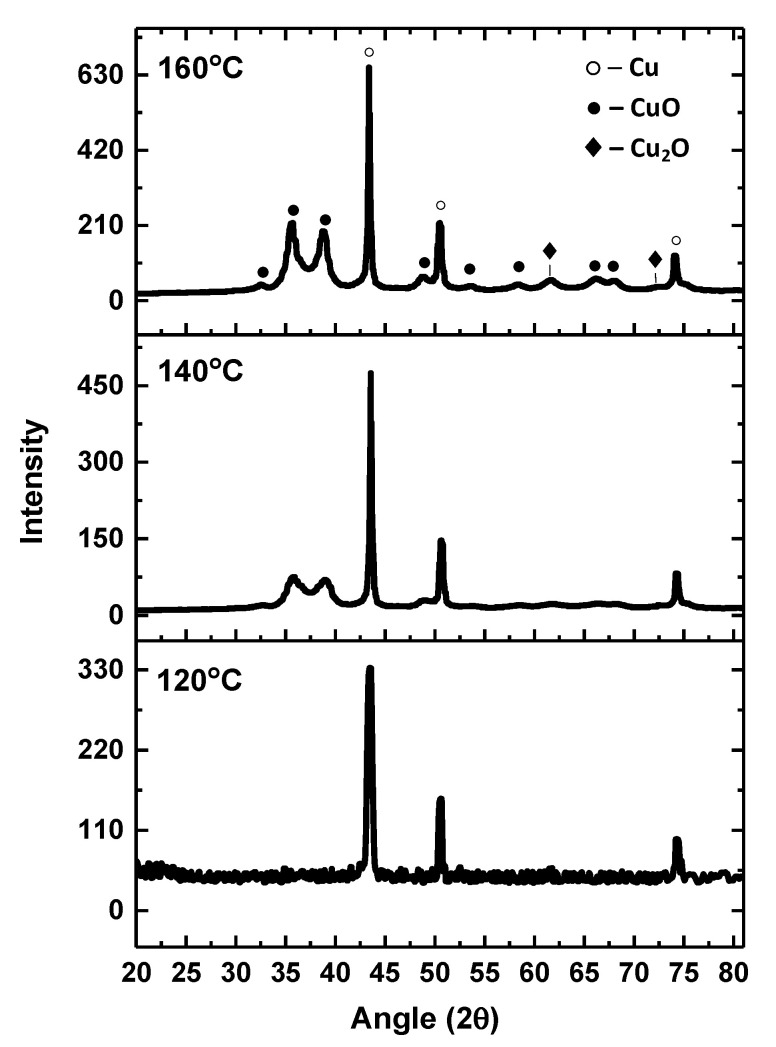
Diffraction patterns of degradation products of the copper dimethylamine complex at various temperatures of processing.

To study the structure and composition of degradation products of the copper dimethylamine complex, we obtained X-ray diffraction patterns of the complexes processed at 120, 140, and 160 °C for 10 min and they are shown in Figure 5.

As can be seen from the diffraction patterns, an increase in the temperature of processing of the copper complex leads to a noticeable increase in the proportion of by-products in the form of copper oxides, which strongly hinder mutual diffusion of Cu atoms between the particles and reduce the formation of percolation paths, and it leads to low conductivity of printed patterns. At a temperature of 120 °C, copper peaks appear clearly. Oxide peaks are hardly observed, however, there appears to be a noisier character of the background curve, which is probably due to the content of residual copper complex in the form of amorphous inclusions. Thus, a temperature of 120–130 °C can be considered optimal for post-processing of the ink based on the dimethylamine complex.

The ink was prepared by dissolving dimethylamine complex in dimethyl sulfoxide at a concentration of 300 mg/mL.

**Figure 6:**
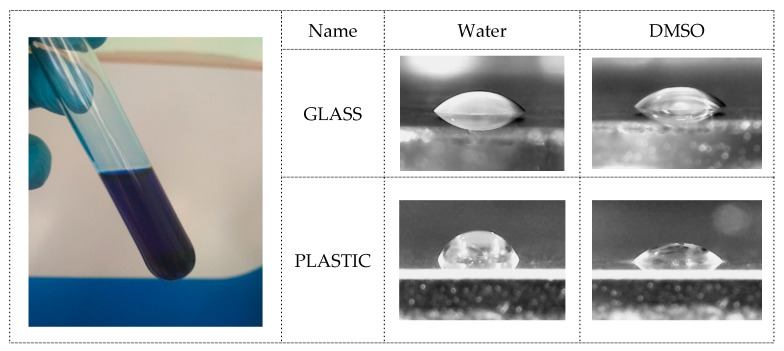
(**Left**): solution of complex compound in dimethyl sulfoxide. (**Right**): microphotographs of drops of water and DMSO on glass and plastic substrates.

The choice of the solvent is conditioned by a number of its advantages, including inactivity in relation to the ink components, the optimal boiling point (190 °C) ensuring stability preventing premature ink drying, and viscosity of 2 mPa·cm (millipascal per centimeter) being optimal for inkjet technology. In addition, we investigated the degree of wetting of glass and plastic surfaces (Figure 6), which is crucial in the printing process, ensuring continuity of the pattern when printing small parts, as well as adhesion of the formed material to the substrate.

The measurements show that dimethyl sulfoxide exhibits the glass surface wetting ability comparable to that of water, due to a certain polarity of the molecules in its composition, while the plastic surface wetting ability is noticeably different—the wetting angle of DMSO on plastic is noticeably smaller than that of water. This effect is conditioned by the affinity of the solvent and the substrate.

### 3.2. Realization of Copper Films on Flexible Substrates

**Figure 7:**
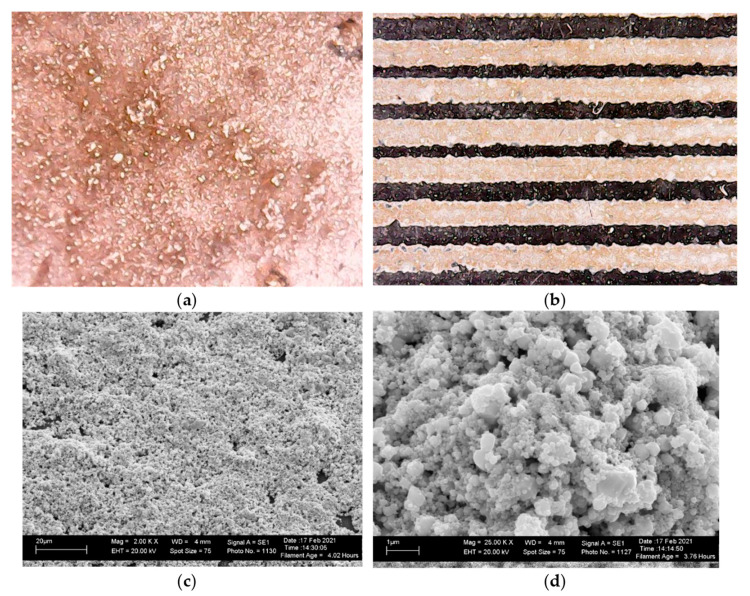
Microphotograph of the surface of the copper layer (**a**) and conductive paths on the surface of the flexible polymer substrate (**b**) and SEM images heated at 120 °C copper layers (**c**,**d**).

The ink was printed using an EPSON 110 inkjet printer. Before use, the ink was pre-filtered with a syringe filter with a pore size of 0.5 μm. Printing was carried out on the surface of a flexible polymer film, and it became possible at a rather low post-processing temperature of the printed ink. Figure 7a shows a microphotograph of the surface of formed copper, which is characterized by a sufficiently high density. On the right there (Figure 7b) are conductive paths 200 µm wide.

The scanning electron microscope (SEM) images (Figure 7c,d) show the microstructure of copper particles formed as a result of thermal decomposition of copper ink. The sizes of particles vary from 100 to 1000 nm. There is a porosity of the formed structures, due to the gaseous reaction products released during the chemical transformation of the ink components. At the same time, the structure is quite dense, and the particles are presented in the form of agglomerates that fused together and provide high electrical conductivity of the printed layers, which is a good indicator for the films annealed at 120 °C.

**Figure 8:**
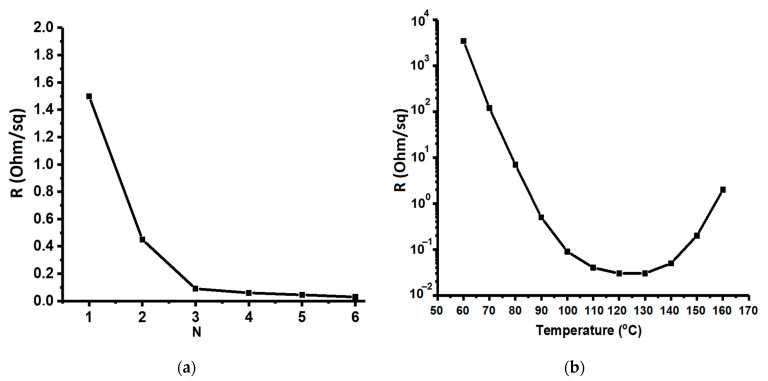
Conductivity versus the number of printing passes (**a**) and temperature of processing of the printed ink (**b**).

The main functional characteristic of copper-based printed layers is conductivity, and its values are presented in Figure 8. The left curve shows the dependence of conductivity on the number of printing passes of the inkjet printer and, as one can see from this figure, the first two passes cause a noticeable decrease in the film resistance, due to an increase in continuity of the copper layer on the substrate surface. An increase in the number of printing passes increases the density and thickness of the layers formed.

The right curve shows conductivity versus post-processing temperature of the printed ink. At low temperatures (60–70 °C), resistance is high—about 2–3 powers of Ohm per square. At these temperatures, ink can be found in the form of complex compounds and exhibit predominantly ionic conductivity. As the temperature increases, there is a noticeable decrease in resistance associated with the appearance of electronic conductivity of the formed metal. However, an increase in temperature above 130–140 °C is accompanied by an increase in resistance of the formed layers, due to an increase in the proportion of by-products in the form of copper oxides, which greatly reduce the number of percolation paths, and it leads to low conductivity of the printed patterns.

## 4. Conclusions

In the course of the research, we obtained the ink based on copper-containing organometallic compounds for the formation of conductive elements of circuits by ink-jet printing. The process of copper reduction from the organometallic complex and the growth of copper grains begins at temperatures above 110 °C and finishes completely at 130 °C, which is acceptable for the formation of electrically conductive paths on most of flexible polymeric materials. As an example, thin conductive copper paths were formed on a PET plastic film using a standard EPSON 110 inkjet printer.

## Figures and Tables

**Table 1:** Thermodynamic potentials of concurrent reactions.

Concurrent Reactions	Δ_r_H°, H	Δ_r_G°, H
Cu^2+^ + 2NH_3_ + 2H_2_O = Cu(OH)_2_ + 2NH_4_^+^	−0.547	−0.516
Cu^2+^ + 2(CH_3_)_2_NH + 2H_2_O = Cu(OH)_2_ + 2(CH_3_)_2_NH_2_^+^	−0.604	−0.574
Cu^2+^ + 2NH_3_ + 2H_2_O = [Cu(H_2_O)_2_(NH_3_)_2_]^2+^	−0.537	−0.483
Cu^2+^ + 2(CH_3_)_2_NH + 2H_2_O = [Cu(H_2_O)_2_((CH_3_)_2_NH)_2_]^2+^	−0.565	−0.503
Cu^2+^ + 2NH_3_ + 2NH_4_^+^ + 2OH^−^ = Cu(NH_3_)_4_^2+^ + 2H_2_O	−1.168	−1.116
Cu^2+^ + 2(CH_3_)_2_NH + 2(CH_3_)_2_NH_2_^+^ + 2OH^−^ = Cu((CH_3_)_2_NH)_4_^2+^ + 2H_2_O	−1.245	−1.179

## Data Availability

Data are available from the authors upon reasonable request.

## Supplementary Materials

The following are available online at https://www.mdpi.com/article/10.3390/ma14092218/s1, Table S1. Images of scanning electron microscopy for the samples tested using the EDX analysis.
