# Supplementary material for: Water-Soluble Copper Ink for the Inkjet Fabrication of Flexible Electronic Components
Source: Materials (Basel). 2021 Apr 26;14(9):2218. doi: 10.3390/ma14092218 (PMC8123473; doi:10.3390/ma14092218)
Supplement: Supplementary file 1 [file materials-14-02218-s001.zip › materials-1143581-supplementary.docx]

Supporting Information

Water-Soluble Copper Ink for the Inkjet Fabrication of Flexible Electronic Components

Nabi S. Shabanov ^1,2^, Kamil Sh. Rabadanov ^1^, Sagim I. Suleymanov ^1^, Akhmed M. Amirov ^1^, Abdulgalim B. Isaev ^2^, Dinara S. Sobola ^2,3,4,^*, Eldar K. Murliev ^1^ and Gulnara A. Asvarova ^1^

^1^ Analytical Center for Collective Use, Dagestan Federal Research Centre of the Russian Academy of Sciences, 367001 Makhachkala, Russia; shabanov.nabi@yandex.ru (N.S.S.); rksh@mail.ru (K.S.R.);
s.sagim.i@yandex.ru (S.I.S.); aamirov@mail.ru (A.M.A.); murliev@mail.ru (E.K.M.);
konfetka080467@mail.ru (G.A.A.)

^2^ Department of Inorganic Chemistry and Chemical Ecology, Dagestan State University, St. M. Gadjieva 43-a, Dagestan Republic, 367015 Makhachkala, Russia; abdul-77@yandex.ru

^3^ Department of Ceramics and Polymers, Faculty of Mechanical Engineering, Brno University of Technology, Technická 2, 616 69 Brno, Czech Republic

^4^ Department of Physics, Faculty of Electrical Engineering and Communication,
Brno University of Technology, Technická 2848/8, 616 00 Brno, Czech Republic

***** Correspondence: sobola@vutbr.cz

**Table S1.** Images of scanning electron microscopy for the samples tested using the EDX analysis. .

|  |  | **Sample** |  |
| --- | --- | --- | --- |
| Т, ^о^С | Cu(OOCH)_2_ | [Cu(NH_3_)_2_](OOCH)_2_ | [Cu(C_2_H_6_NH)_2_](OOCH)_2_ |
| 150 | 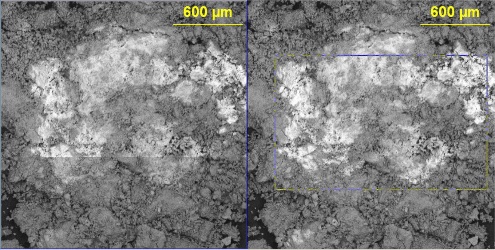 | 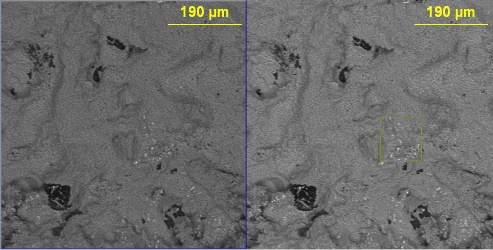 | 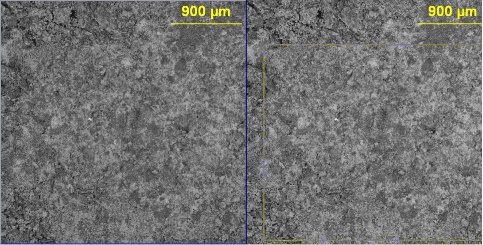 |
| 130 | 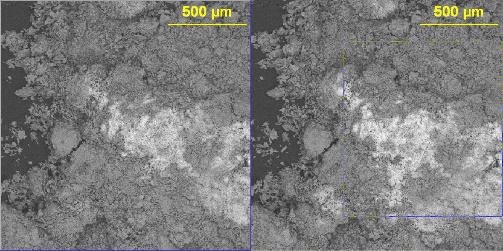 | 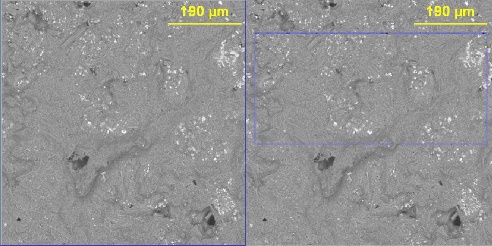 | 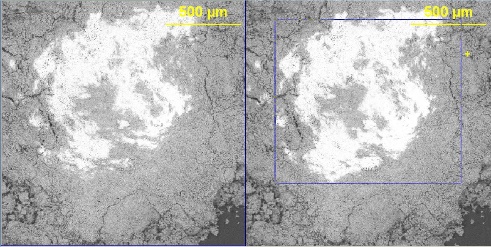 |
| 110 | 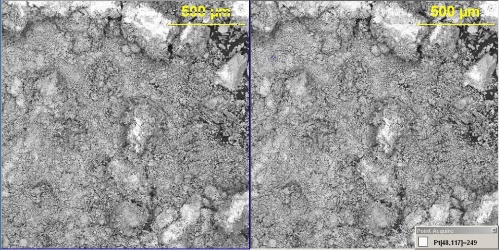 | 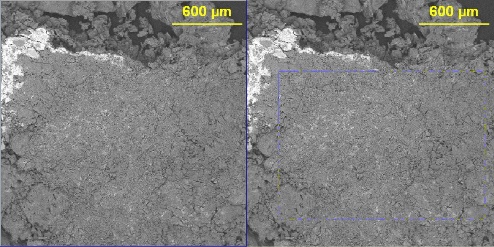 | 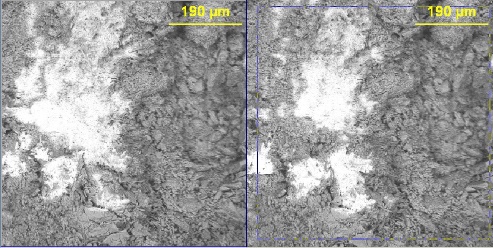 |
| 25 | 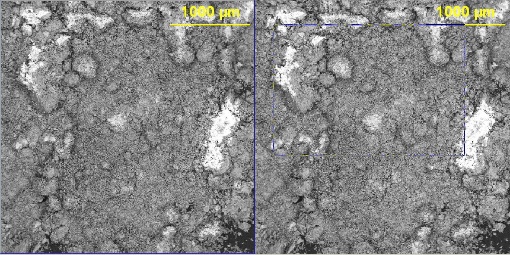 | 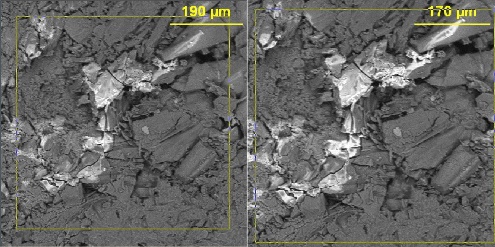 | 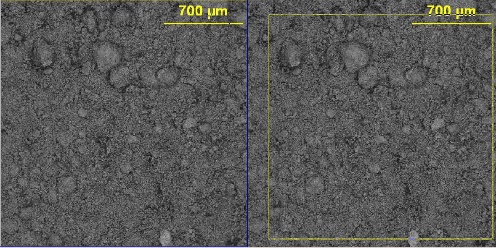 |
