# Supplementary material for: Water-Soluble Copper Ink for the Inkjet Fabrication of Flexible Electronic Components
Source: Materials (Basel). 2021 Apr 26;14(9):2218. doi: 10.3390/ma14092218 (PMC8123473; doi:10.3390/ma14092218)
Supplement: Supplementary file 1 [file materials-14-02218-s001.zip › materials-1143581-supplementary.pdf]

materials

MDPI

Supporting Information

Water-Soluble Copper Ink for the Inkjet Fabrication of Flexible Electronic Components

Nabi S. Shabanov [1,2], Kamil Sh. Rabadanov [1], Sagim I. Suleymanov [1], Akhmed M. Amirov [1], Abdulgalim B. Isaev [2], Dinara S. Sobola [2,3,4,*], Eldar K. Murliev [1] and Gulnara A. Asvarova [1]

1 Analytical Center for Collective Use, Dagestan Federal Research Centre of the Russian Academy of Sciences, 367001 Makhachkala, Russia; shabanov.nabi@yandex.ru (N.S.S.); rksh@mail.ru (K.S.R.); s.sagim.i@yandex.ru (S.I.S.); aamirov@mail.ru (A.M.A.); murliev@mail.ru (E.K.M.); konfetka080467@mail.ru (G.A.A.)
2 Department of Inorganic Chemistry and Chemical Ecology, Dagestan State University, St. M. Gadjieva 43-a, Dagestan Republic, 367015 Makhachkala, Russia; abdul-77@yandex.ru
3 Department of Ceramics and Polymers, Faculty of Mechanical Engineering, Brno University of Technology, Technická 2, 616 69 Brno, Czech Republic
4 Department of Physics, Faculty of Electrical Engineering and Communication, Brno University of Technology, Technická 2848/8, 616 00 Brno, Czech Republic
* Correspondence: sobola@vutbr.cz

Table S1. Images of scanning electron microscopy for the samples tested using the EDX analysis. .

	Sample		
T, °C	$Cu(OOCH)_2$	$[Cu(NH_3)_2](OOCH)_2$	$[Cu(C_2H_6NH)_2](OOCH)_2$
150	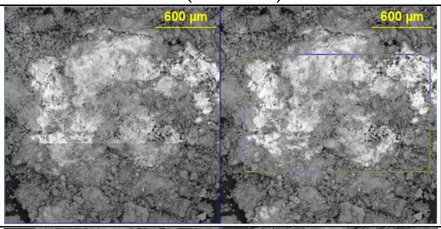	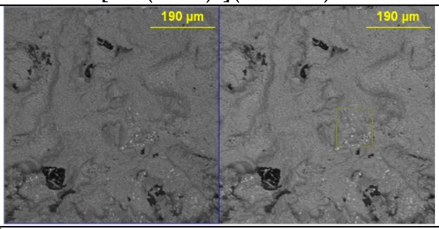	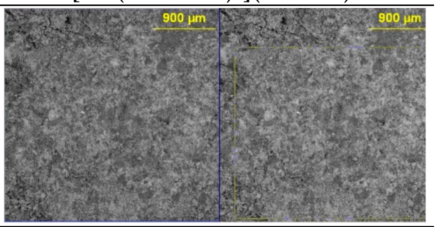
130	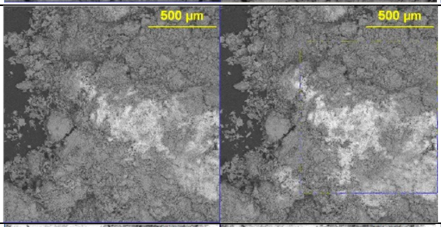	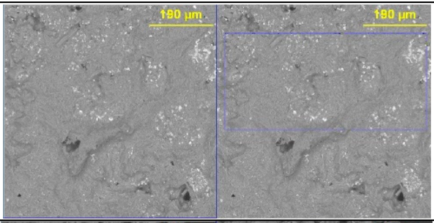	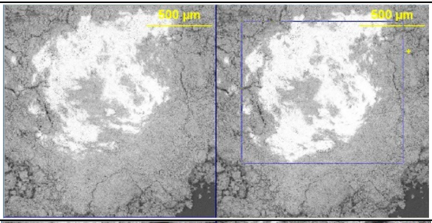
110	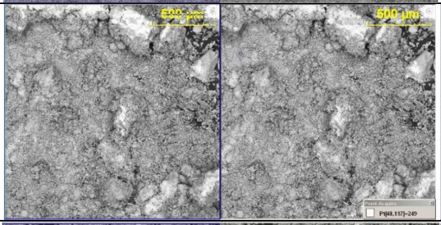	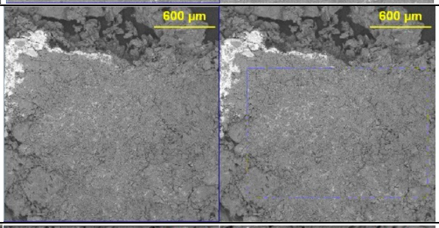	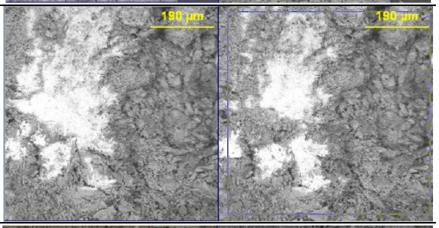
25	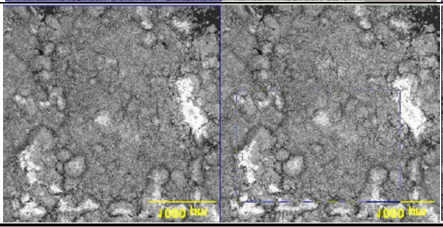	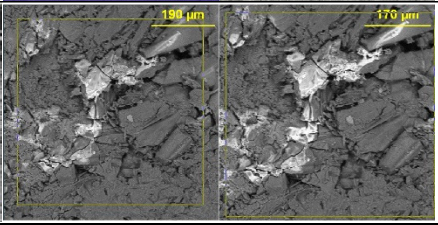	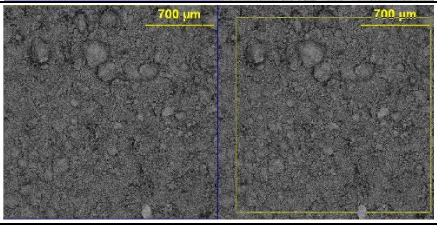
